# Towards a Better and Harmonized Education in Antimicrobial Stewardship in European Veterinary Curricula

**DOI:** 10.3390/antibiotics10040364

**Published:** 2021-03-30

**Authors:** Carmen Espinosa-Gongora, Lisbeth Rem Jessen, Oliver James Dyar, Alain Bousquet-Melou, Bruno González-Zorn, Céline Pulcini, Giovanni Re, Stefan Schwarz, Dorina Timofte, Pierre-Louis Toutain, Luca Guardabassi, The PREPARE-VET Working Group, ESCMID Study Group for Veterinary Microbiology (ESGVM), ESCMID Study Group for Antimicrobial stewardshiP (ESGAP)

**Affiliations:** 1 Department of Veterinary and Animal Sciences, University of Copenhagen, 1870 Frederiksberg C, Denmark; 2 Department of Veterinary Clinical Sciences, University of Copenhagen, 1870 Frederiksberg C, Denmark; lrmj@sund.ku.dk; 3 Department of Global Public Health, Karolinska Institutet, 17165 Stockholm, Sweden; oliver.dyar@ki.se; 4 INTHERES—Innovations Thérapeutiques et Résistances, École Nationale Vétérinaire de Toulouse, 31076 Toulouse, France; alain.bousquet-melou@envt.fr (A.B.-M.); pltoutain@wanadoo.fr (P.-L.T.); 5 Antimicrobial resistance Unit, VISAVET Health Surveillance Centre, Veterinary School, Complutense University of Madrid, 28040 Madrid, Spain; bgzorn@ucm.es; 6 APEMAC, Université de Lorraine, F-54000 Nancy, France; celine.pulcini@univ-lorraine.fr; 7 Infectious Diseases Department, Université de Lorraine, CHRU-Nancy, F-54000 Nancy, France; 8 Department of Veterinary Sciences, University of Turin, I-10095 Turin, Italy; giovanni.re@unito.it; 9 Institute of Microbiology and Epizootics, Freie Universität Berlin, 14163 Berlin, Germany; stefan.schwarz@fu-berlin.de; 10 Institute of Infection, Veterinary and Ecological Sciences, Department of Anatomy Physiology and Pathology, University of Liverpool, Leahurst Campus, Cheshire CH64 7TE, UK; d.timofte@liverpool.ac.uk; 11 The Royal Veterinary College, University of London, Hawkshead Campus, Hatfield AL9 7TA, UK

**Keywords:** antimicrobial stewardship, veterinary medicine, one health, veterinary curriculum, education, antimicrobial resistance, questionnaire, preparedness

## Abstract

Education in antimicrobial stewardship (AMS) in veterinary medicine is essential to foster responsible antimicrobial use and control of antimicrobial resistance (AMR) in animals. AMS is listed by the EU and international organizations among the basic ‘Day One Competences’ required of veterinary students upon graduation. Our aim was to evaluate the quality of education of European veterinary students in AMS. We distributed a 27-item survey addressing the perceptions of preparedness and acquired skills on key topics related to AMS to final-year veterinary students in Europe. We collected 3423 complete answers from 89 veterinary schools in 30 countries. Selection of treatment strategies and awareness of emerging AMR problems were markedly different between countries. Overall, only one in four students was familiar with guidelines for antimicrobial use. The students perceived a medium-high impact of veterinary antimicrobial use on AMR in humans. Notably, 75% of the students felt the need for improved teaching on AMS, half of which also demanded more teaching on general antimicrobial therapy. Our results highlight several possible strategies to improve the quality of education, ranging from a better link between clinical rotations and the theory taught in pre-clinical modules, to a more effective introduction into best practices for antimicrobial use.

## 1. Introduction

Veterinarians and medical doctors stand in the frontline of the battle against antimicrobial resistance (AMR) and face the common challenge of prescribing and monitoring the use of antimicrobials agents prudently. Antimicrobial stewardship (AMS) is a coherent set of actions which promote using antimicrobials in ways that ensure sustainable access to effective therapy for all who need them [1].

Education in AMS is essential to foster responsible antimicrobial use in both human and veterinary medicine. Current competences described in the curricula of veterinary programs in Europe rely on different pieces of EU legislation, and guidelines by the World Organization for Animal Health (OIE), the European Commission, the Federation of Veterinarians of Europe, the European Association of Establishments for Veterinary Education, and the World Health Organization. The European Coordinating Committee on Veterinary Training (ECCVT) has recently summarized these sources in their Annex 2. List of subjects and Day One Competences [2]. Among these competences, it is stated that new veterinary graduates need to understand (i) the aetiology, pathogenesis, diagnosis and treatment of animal diseases, (ii) medicines legislation and guidelines on responsible use of antimicrobial agents, and (iii) the principles of disease prevention and the promotion of health and welfare. Altogether these professional demands represent the fundamentals of AMS, and the minimum standard required for veterinary education in this field. Therefore, these very general competences need to be adapted into the detailed training at the national level, often supported by national professional bodies, but there is a gap in the literature to guide such development. Previous studies conducted among veterinary students in South Africa [3] and Australia [4] revealed that they were not satisfied about the quality and quantity of education in AMS received during their veterinary curricula, whereas respective large-scale European data is missing.

Inspired by the PREscriber Perspectives on Antibiotic use and Resistance Education (Student-PREPARE) survey conducted in 2015 by the European Society of Clinical Microbiology and Infectious Diseases (ESCMID) study group for AMS (ESGAP) among final-year medical students in Europe [5], PREPARE-VET was developed as a joint initiative by the ESCMID study group for veterinary microbiology (ESGVM) and ESGAP to evaluate the need for further education of European veterinary students in AMS.

## 2. Results

### 2.1. Participation in the Survey

Collaborators from 31/33 countries and 97/104 veterinary schools provided consent to participate in the study. Eight of the 97 schools were further excluded due to either absence of data collection (*n* = 3), participation below 10% (*n* = 4) or unknown number of eligible students (*n* = 1), leading to 30 countries and 89 schools finally enrolled in the study (Supplementary Materials Figure S1). A total of 36 country coordinators (CCs) were in charge of survey distribution in the 30 countries (Croatia, France, Slovenia, Switzerland, and Turkey had two coordinators each, Italy had three, and UK and Romania shared one).

We collected 5567 answers, of which 3423 were used for data analysis. Answers were excluded if the participation in a particular school was below 10% (four schools, *n* = 19 answers), if participation could not be estimated (due to lack of information on the total number eligible students) (one school, *n* = 12 answers), if the identity of the veterinary school was not indicated (*n* = 12 answers), or due to incompleteness (*n* = 2101 answers, including 667 students that were not enrolled in the final year of their veterinary education). Average participation among the 89 veterinary schools was 45% (range 12–100%, median 34%). The list of veterinary schools included in the final analysis, the number of complete answers per school and participation are provided in Table S1.

### 2.2. Participants’ Profiles

Students whose responses were included in the final analysis were represented by a higher proportion of women (65%), 25 year olds (median; IQR = 2), and who intended to specialise in companion animal/equine medicine (46%), food animal medicine (27%), other (14%), or were undecided about future specialization (13%). When asked about their overall performance as a veterinary student on a scale from 1 to 10 (10 representing a top student, 5 an average student, and 1 a student at the bottom of the rank), most students ranked themselves 7 (median; IQR = 2). At the time of completing the survey, 63% of the students had already completed the clinical rotations as part of their veterinary education, 27% were enrolled in the rotations in that moment, 8% had not started yet, and 2% reported not having clinical rotations included in their veterinary curriculum. The latter was considered an error during completion or interpretation of the survey since nearly all students from the same schools reported inclusion of clinical rotations in their curricula.

### 2.3. Students’ Perception of Preparedness

**Figure 1:**
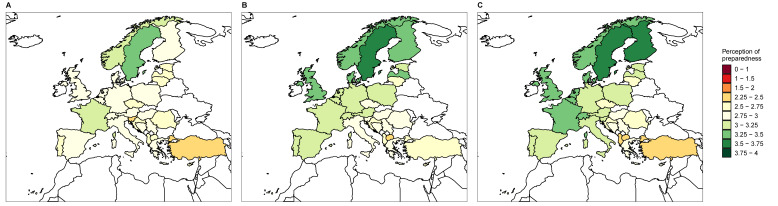
Average perception of preparedness in topics related to pharmacology of antimicrobial agents (**A**), clinical use of antimicrobials agents (**B**), and antimicrobial resistance (**C**). Perception of preparedness measures as “Well prepared” (4), “Sufficiently prepared” (3), “Poorly prepared” (2), “Not at all prepared” (1), and “I haven’t received any teaching or training” (0).

The average students’ perception of preparedness was below level 3 (“Sufficiently prepared”) in all three fields of knowledge evaluated, with weighted means of 2.73, 2.94, and 2.93, in pharmacology of antimicrobial agents, clinical use of antimicrobial agents, and AMR, respectively. There were differences between countries in all three fields of knowledge (Figure 1). Only four out of the 15 questions about perception of preparedness scored above “Sufficiently prepared”, i.e., Differential diagnosis to bacterial infection (i.e., fungi, viruses, parasites, aseptic inflammation) (score = 3.01), Antimicrobial susceptibility testing (AST) (score = 3.03), Infection control and prevention practices (score = 3.1), and Impact of AMR on public health (score = 3.29) (Figure S2). Based on pairwise Wilcoxon test, average perception of preparedness in all three fields was significantly higher in students that had already completed their clinical rotations (*p* < 0.05) (Figure S3). Linear mixed models confirmed the positive impact of having completed the clinical rotations on average perception of preparedness in the field of clinical use of antimicrobial agents. These and other variables significantly influencing the average perception of preparedness of students are shown in Table 1. Final models are shown in Table S2.

### 2.4. Assessment of Students’ Preparedness

**Figure 2:**
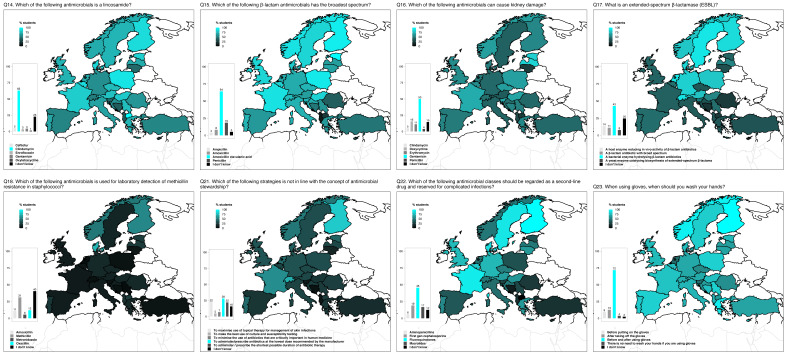
Percentage of final-year veterinary students selecting the correct answer to questions related to antimicrobial agents and infection control in veterinary medicine. Q17 should be interpreted carefully in Austria, Germany, and Switzerland due to an error in the German-language version of the survey (two correct options for German-speaking students instead of one). Bar plots show the percentage of students selecting each of the answers available (bottom legends), and correct answers are assigned the cyan colour. Maps display the percentage of students that selected the correct answer (top-left legends). Bar plots may display added percentages above or below 100% due to rounding of the values.

In general, the students performed better at topics related with general knowledge and clinical use of antimicrobial agents (e.g., nomenclature, spectrum of activity, first/second-line antimicrobial agents, infection control), than at topics related to AMR (e.g., emerging resistant pathogens) and AST. More students from North and Central Europe (Austria, Germany, The Netherlands, Poland, Scandinavia, and Switzerland) provided correct answers to the meaning of extended-spectrum β-lactamases (ESBL) (Q17) compared to students from other regions. Danish, Finish, French, and Swedish students were best at identifying fluoroquinolones as a second-line drug reserved for complicated infections (Q22). These and other geographical trends in this section are shown in Figure 2. The knowledge score estimated from questions Q14–18, 21–23 was positively correlated with all three fields of perception of preparedness (r ≥ 0.3).

**Figure 3:**
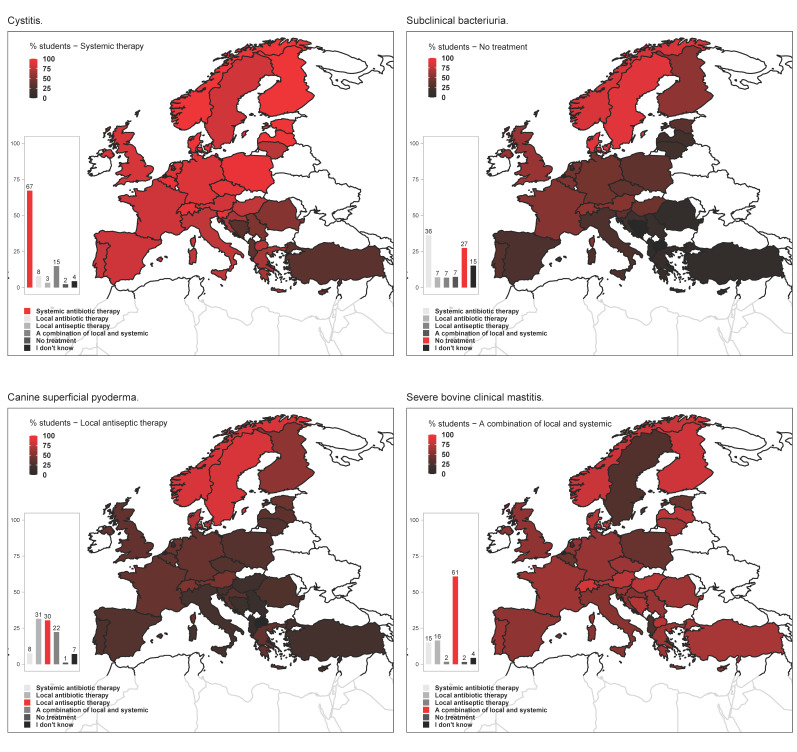
Treatment of choice by final year veterinary students in four clinical cases presented to them in question 20 of the survey: “Please indicate which treatment strategy you were taught to apply for the following infections”. Bar plots show the percentage of students selecting each of the answers available (bottom legends). Maps display the percentage of students that selected the answer specified in the top-left legends. Bar plots may display added percentages above or below 100% due to rounding of the values.

In the questions simulating performance in clinical scenarios, more than 60% of the students were able to determine the most probable aetiology of equine strangles and upper respiratory tract infections in cats, whereas less than 50% assigned *Escherichia coli* to the most probable cause of canine urinary tract infections, and less than 40% (essentially only students from North and Central European countries) selected *Lawsonia intracellularis* as the most common cause of ‘greasy diarrhoea’ in 20–30 kg pigs and weight loss among other listed pathogens (*E. coli*, *Streptococcus* spp., *Pseudomonas* spp. or virus) (Figure S4). Systemic antimicrobial therapy for treatment of canine cystitis and a combination of local and systemic antimicrobial therapy for treatment of severe bovine clinical mastitis were the most popular choices in question Q20 (weighted averages 67% and 61%, respectively). With regards to severe bovine clinical mastitis, most Swedish students (>70%) selected systemic therapy in contrast to a combination of local and systemic therapy picked by the majority of students in other countries. Similarly, the most commonly elected choice of treatment for subclinical bacteriuria was systemic therapy, although students from Norway, Sweden and Denmark mainly chose not to treat. For treatment of canine superficial pyoderma, most students from Scandinavian countries selected local antiseptic therapy, whereas most students in the other countries selected either local antibiotic therapy, or a combination of local and systemic therapy (Figure 3).

**Figure 4:**
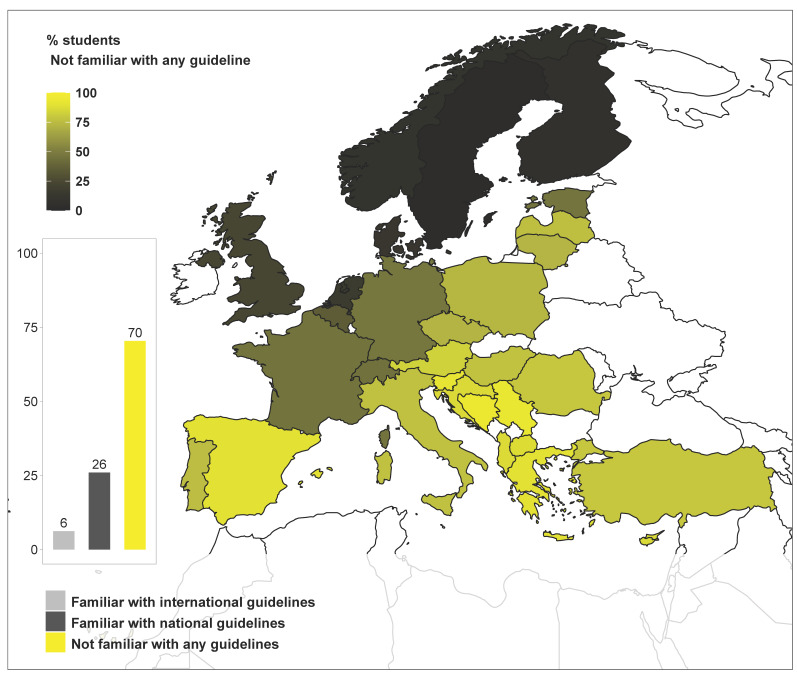
Percentage of final-year veterinary students that are not familiar with national or international guidelines for rational antimicrobial use, as reported in question 24 of the survey: “Are you familiar with any practice guidelines for rational antimicrobial use?”. The bar plot shows the percentage of students selecting each of the answers available (bottom legend). The map displays the percentage of students that selected the answer: “Not familiar with any guideline” (top-left legend). Bar plots may display added percentages above or below 100% due to rounding of the values.

Out of 3423 students, 2410 (70%) reported not being familiar with any clinical guideline for rational antimicrobial use. This large number of students was unevenly distributed in Europe, with most originating from countries in the South and East (Figure 4).

### 2.5. Students’ Perception of the Impact of Veterinary Antimicrobial Use

Students were asked for their opinion about the relative contribution of veterinary use of antimicrobial agents to clinical problems caused by resistant bacteria in humans. Overall, 43% of students believed the relative contribution of veterinary antimicrobial use was Medium (10–20%) (score 2) (Figure S5). Only seven countries recorded lower perceived impact: Norway (score 1.4), Denmark (1.6), The Netherlands (1.7), Switzerland (1.7), United Kingdom (1.7), Germany (1.8), and Albania (1.9); while the three countries with highest values (highest perceived impact) were Croatia (2.5), Serbia (2.5), and Portugal (2.6).

### 2.6. Impact of Teaching Methods on Students’ Perceived Preparedness and Knowledge

The models built to analyze data from Section 2.3 and Section 2.4 were used to study the impact of different teaching methods on the students’ perception of preparedness and on the students’ AMS knowledge, respectively. Linear mixed models showed that frequent teaching by lectures and discussions of clinical cases had a positive impact on the average perception of preparedness in the field of clinical use of antimicrobial agents and AMR, but not on the knowledge score. In contrast, clinical rotations had a significant positive effect on the average perception of preparedness only in the field of clinical use on antimicrobial agents and on the knowledge score (Table 1). Teaching methods had no significant associations with overall antimicrobial sales at country level.

### 2.7. Satisfaction with the Received Education in AMS

**Figure 5:**
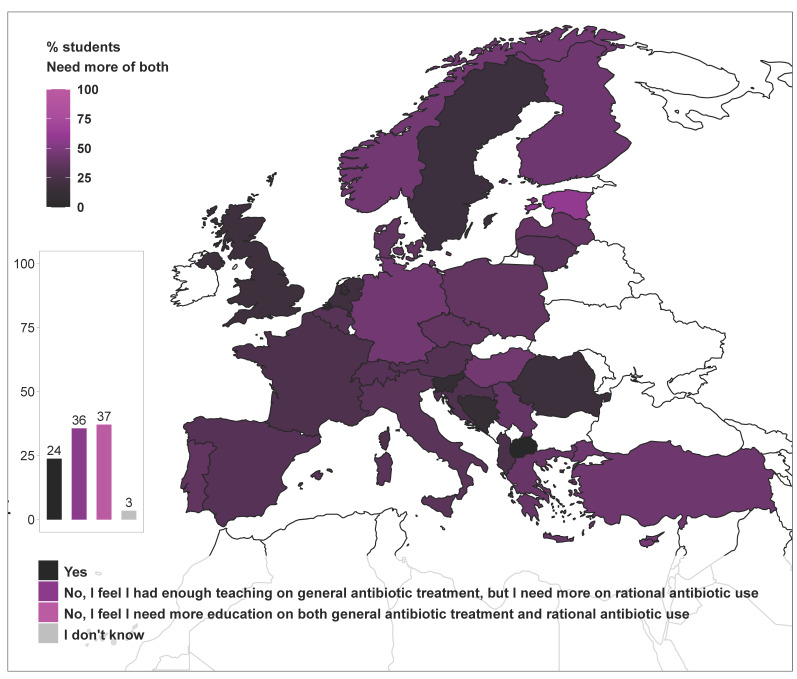
Satisfaction of final-year veterinary students about their knowledge in use of antimicrobial agents, as reported in question 27 of the survey: “Overall, do you think you receive adequate teaching to face antimicrobials and resistance issues in clinical practice?”. The bar plot shows the percentage of students selecting each of the answers available (bottom legend). The map displays the percentage of students that selected the answer: “No, I feel I need more education on both general antibiotic treatment and rational antibiotic use” (top-left legend). Bar plots may display added percentages above or below 100% due to rounding of the values.

Almost 75% of the students felt they needed more teaching on rational antimicrobial use, of which approximately half also demanded improved teaching on general knowledge about antimicrobial therapy (Figure 5). Students satisfied with the teaching they received displayed higher perception of preparedness in all study fields and higher knowledge score (Table 1).

### 2.8. Trends in Antimicrobial Sales

Final linear models fitting antimicrobial sales data were reduced due to collinearity between some of the variables included. Details of final, non-collinear models are shown in Table S2. Lower overall antimicrobial sales were significantly associated to a higher proportion of students selecting systemic therapy for treatment of cystitis (Table 1).

## 3. Discussion

The survey revealed a clear demand by students for more and better teaching in AMS and identified specific topics that should be more effectively covered by European veterinary curricula. On average, students’ perception of preparedness remained under the level of “Sufficiently prepared” in questions related to pharmacology, clinical use, and AMR. Among these three fields, students felt least confident in pharmacology-related questions, similar to students in South African and Australian veterinary schools in previous studies [3,4]. Aside from their low perception of preparedness, students often failed in AMR-related theoretical questions. For example, a great proportion of students were not able to i) define ESBL (despite this being one of the most common multi-drug resistance determinants of public and animal health concern [6]), ii) discern fluoroquinolones from a list of first-line antimicrobial agents (similar to the results of a recent study in Serbia and Croatia [7]), or iii) select the antimicrobial surrogate drug used for detection of methicillin resistance in staphylococci (oxacillin).

Students failed to answer correctly specific questions addressing AMS practices, such as Q21: “Which of the following strategies is NOT in line with the concept of AMS?”. Similar trends were observed in previous studies conducted on South African veterinary students and European junior medical doctors [3,5,8,9]. Just above one fourth of the students (28%) selected the correct answer “To prescribe antibiotics at the lowest dose recommended by the manufacturer”, a discouraged practice due to the risk of selecting AMR and reducing clinical efficacy [8]. One of the wrong answers “To administer the shortest possible duration of therapy” was relatively popular (selected by 22% the students), possibly because historically, it was recommended to continue therapy well beyond clinical improvement in order to reduce relapses, and due to the belief that prolonged therapy could reduce the risk of AMR development. However, the current evidence-based recommendation is to cease treatment after resolution of clinical signs to avoid unnecessary selective pressure [9]. The authors retrospectively noticed certain ambiguity in one of the other options presented to the students “To maximize the use of topical therapy for management of skin infections” (selected by 22% of the students), which could have been misunderstood without specifying that we meant maximizing the use of topical therapy instead of systemic therapy. We therefore advice results from this question should be interpreted with caution.

There was a direct correlation between perception of preparedness and the estimated knowledge score, indicating that students’ perception as investigated in several previous surveys on AMS in human and veterinary medicine [4,5,8] can be used as a useful proxy to estimate actual preparedness. Despite the statistical association, we found a few discrepancies between questions addressing students’ perceptions of preparedness and actual knowledge. First, a large proportion of students reported being at least sufficiently prepared on the impact of AMR on public health (83%) and on emerging zoonotic or veterinary multidrug resistant (MDR) pathogens (63%), but only 43% were able to define ESBL. Second, 69% of students felt at least sufficiently prepared to select an antimicrobial drug and a regimen of therapy, but 52% failed to indicate fluoroquinolones as second-line drugs that should be reserved for management of complicated infections. Previous studies with medical students and junior doctors have discussed possible reasons that may impact their perception of preparedness, such as cultural factors and prevalence of antimicrobial resistance in a country [5]. The same factors might have influenced the geographical differences observed between students from diverse European regions.

With regard to assignment of the most common aetiology to basic clinical cases, there was an apparent lack of awareness about the role of *L. intracellularis* in porcine diarrhoea in weaned pigs in Southern European countries, which does not seem to be justified by difference in the disease prevalence between North and South [10]. While at least 50% of students in 26/30 countries selected the right option for treatment of cystitis (systemic therapy), students providing the correct answer to treatment of subclinical bacteriuria (no treatment) originated from only 9/30 countries (Austria, Belgium, Denmark, Finland, France, Norway, Sweden, Switzerland, United Kingdom). The most popular answer (36.2%) to treat subclinical bacteriuria was systemic antimicrobial therapy, which is not recommended except for special and well-defined clinical scenarios according to current guidelines (e.g., animals at high risk of ascendant or systemic infection) [11]. Another marked geographical trend was that only students from Scandinavia and Switzerland predominantly selected local antiseptic therapy for treatment of canine superficial pyoderma, which is currently recommended in view of the demonstrated efficacy and lower AMR selective pressure of antiseptic products as compared to systemic antimicrobial agents [12,13]. At least half of the surveyed students in 25/30 countries would treat severe bovine mastitis with a combination of systemic and local antimicrobial treatment. Sweden stood out with a majority of students selecting systemic therapy in line with a recent Swedish study showing a change in management of bovine mastitis in Sweden over the last years towards sole parenteral therapy, as recommended in the national guidelines unless *Staphylococcus aureus* or *Streptococcus agalactiae* are confirmed [14].

An interesting finding was that teaching strategies were positively associated to students’ perception of preparedness and to actual students’ knowledge assessed by the questionnaire, but not to antimicrobial sales. This could be due to the fact that AMS is still at the embryonic stage of its development in veterinary medicine, and consequently not sufficiently covered by veterinary curricula. Our results suggest that frequent lectures, and discussions of clinical cases are important to ensure student preparedness in this field. Of note, at least 50% of veterinary students from 19 countries reported not being familiar with any national or international practice guidelines for antimicrobial use, which are a cornerstone of AMS. Among the remaining 11 countries, five still reported rather high proportions (at least 1/3) of students reporting not being familiar with these guidelines (Belgium, Estonia, France, Germany, Switzerland), and only the last six (Denmark, Finland, The Netherlands, Norway, Sweden, United Kingdom) displayed a majority of students being familiar with guidelines. This is a regretful sign that students do not receive sufficient teaching on this subject, which should be carefully considered in future revisions of the current veterinary curricula.

Clinical rotations had a positive impact on students’ perception of preparedness on the topic of clinical use of antimicrobial agents, but not on topics related to pharmacology or AMR. Similarly, a previous survey in Australia indicated that veterinary students felt clinical teaching of antimicrobial use was more useful than the pre-clinical teaching learnt in previous years, even though pre-clinical teaching appeared superior in the teaching of appropriate antimicrobial use [4]. From these data it appears that pharmacology and AMR-related topics, which are generally taught as part of courses in pharmacology, microbiology and infectious diseases, are not sufficiently covered during the clinical rotations, where students have the opportunity to put into practice the notions learned during the first years of study on how to prevent AMR by rational antimicrobial use. This suggests that a better coordination between what is taught in the pre-clinical and clinical modules would be desirable.

Finally, with regard to the opinion question about the relative contribution of veterinary antimicrobial use to clinical AMR problems in humans, it is noteworthy that the majority of students showed awareness of the public health risks derived from the use of antimicrobial agents in animals. In fact, the contribution was perceived as *high* (>50%) and *medium* (10–20%) by at least 50% of students from 7 and 14 countries, respectively. None of the remaining options (*low*, *very low*, or *uncertain*) was predominant in any of the countries. Only students from Finland and Sweden seemed to acknowledge the complexity of this question displaying the highest rates of *uncertain* (25 and 20%, respectively). The complexity of this question lies in the fact that a thorough evaluation of the contribution of veterinary use of antimicrobial agents to AMR problems in human medicine requires a one-by-one analysis of the target resistant pathogens and antimicrobial agent, and requires comprehensive epidemiological, quantitative risk assessment and source attribution studies for each specific bug-drug combination. Recent research suggests that this impact might have been overestimated for certain resistant bacteria of high clinical relevance that are increasingly reported in animals, such as ESBL-producing *E. coli* [15,16]. Australian practitioners and veterinary students from Australia and South Africa perceived a moderate contribution of veterinary antimicrobial use to overall AMR problems, which was mostly attributed to intensive animal industries (despite most reports of multi-drug resistant pathogens in animals originating from companion animals) [3,4,6].

Our study is the first to provide European-wide data on AMS knowledge and perceived preparedness of final-year veterinary students, including an extensive list of countries and veterinary schools. A limitation to the study is the uneven veterinary school portray of the countries, in terms of number of schools and number of students enrolled in each school. Although such variability is accounted for in the models using random effects and does not affect the country-based analyses and comparisons, it is possible that the results of the study may be more relevant for the countries that provided the study with larger amounts of data.

## 4. Materials and Methods

### 4.1. Questionnaire Development

In 2017, a PREPARE-VET core study group of experts in AMS, veterinary medicine, public health, microbiology and pharmacology from Denmark, Italy, France, Germany, Romania, Spain, and United Kingdom was established to define a set of desirable learning outcomes in responsible antimicrobial use. The list of learning outcomes was developed based on expert opinion, and review of veterinary school curricula and similar studies previously conducted among final-year medical students [17], and incorporated topics and concepts of specific relevance to veterinary education. The document was iteratively reviewed by the core group members until consensus was reached. The final consensus document is presented in Table S3.

The PREPARE-VET survey was developed using elements from the list of desirable learning outcomes in veterinary AMS. Following revision and approval by all members of the core group, a first version of the survey was piloted among 34 volunteer final-year veterinary students from Denmark, France, Italy, Turkey, and the United Kingdom, who were asked about the level of difficulty and clarity of the questions. The survey was amended based on the comments from the volunteer students, leading to the final survey including 27 questions (Table S4). Questions were divided into six categories: (1) student’s profile; (2) student’s perception of preparedness in AMS; (3) assessment of student’s preparedness; (4) student’s perception of the impact of veterinary antimicrobial use on AMR problems in humans; (5) teaching methods; and (6) overall satisfaction with the received education in AMS. Student’s perception of preparedness (Section 2.3) was evaluated by 15 specific questions within three fields of knowledge, (i) pharmacology of antimicrobial agents, (ii) clinical use of antimicrobial agents, and (iii) AMR. Students scored their perception of preparedness as “Well prepared” (4), “Sufficiently prepared” (3), “Poorly prepared” (2), “Not at all prepared” (1), and “I haven’t received any teaching or training” (0). Questions assessing student’s preparedness (Section 2.4) were formulated to cover key concepts or definitions related to antimicrobial drugs and AMR as well as clinical scenarios to evaluate the student’s competence to control AMR through rational antimicrobial use and prevention of disease transmission. Average relative contribution of veterinary use of antimicrobial agents (Q25) per country was calculated assigning values to each answer: Very low (0), Low (1), Medium (2), High (3), and Uncertain (NA).

The final survey was translated from English into 10 languages (Albanian, Estonian, German, Hungarian, Lithuanian, Norwegian, Polish, Serbian, Spanish, and Turkish) and made available on the online platform SurveyMonkey^®^.

### 4.2. Survey Distribution and Target Population

A network of collaborators in all European countries offering a veterinary graduate degree was established to distribute the survey. First, contact was established with university staff members within the ESGVM network. Where there was a lack of adequate contacts within ESGVM, collaborators were identified within departments of infectious diseases, microbiology or pharmacology using the website of each veterinary faculty. We identified potential collaborators in 33 countries, who were invited to become country coordinators (CC). Subsequently, CCs were requested to establish contact with all veterinary schools in the country and appoint veterinary school coordinators in each.

Students were eligible to participate in the study if they were enrolled in the final year of the veterinary curriculum with active teaching activities. Students that had completed all semesters with teaching activities, but were still enrolled in examinations-only semesters, were also eligible. The survey was distributed between June 2017 and June 2018 using the online platform. The total number of students enrolled in the eligible semesters in each school was requested from CCs to estimate participation rates. Schools collecting answers from at least 10% of the eligible students were included in the final analysis. Students were requested to complete the survey only once. In order to avoid duplicate answers derived from failed attempts to complete the survey, only complete answers to the whole survey (where 100% of questions in the survey were replied to) were included in the analysis.

### 4.3. Ethical Statement

In compliance with the General Data Protection Regulation (GDPR), students were informed at the start of the survey that their participation was voluntary, and that personal data would remain anonymous. All veterinary schools provided consent to participate in the study to their corresponding CC. Ethics approval for participation of students in the survey was obtained from local Veterinary School Committees when needed.

### 4.4. Data Analysis

Data analysis and visualization were performed in R version 3.6.1 [18]. Data inspection was performed prior to statistical analyses using Pearson’s correlation values, scatterplots for inspection of continuous data and boxplots for categorical data. Subsequently, Wilcoxon test, linear mixed models and linear regression were used to analyze our dataset. A summary of research questions and the corresponding statistical methods are shown in Table S5. To reduce the size of models, we created a knowledge score for each student calculated as the percentage of correct answers to eight survey questions (Q14–18, Q21–23) (Q19–20 are not part of the knowledge score due to multiple possible correct answers). For country-level analyses, the knowledge score was calculated as the average percentage of students giving the correct answer to each question. To avoid redundancy in model construction, the variable clinical rotations in the categorical format (Q4) was selected over the frequency format (Q26). In order to explore whether countries that are already implementing better education in AMS had also different patterns in prescription of antimicrobial agents, we analyzed possible associations between students’ preparedness and antimicrobial sales at the country level using data on overall sales of veterinary antimicrobial agents from 2017 (expressed in mg/PCU) exported from the European Surveillance of Veterinary Antimicrobial Consumption (ESVAC) online database [19]. Collinearity was examined in all models using the performance package in R [20]. All average values across countries are reported as weighted arithmetic means to account for the different student population size in each country. All plots were made using ggplot2, ggmap, and dplyr packages in R [21,22].

## 5. Conclusions

The results of our survey demonstrate that veterinary students in Europe are aware of the risks to public health posed by antimicrobial use in animals. On the other hand, the results also highlight a few important areas that could be better addressed by veterinary curricula to optimize veterinary education in AMS. These areas include but are not limited to (i) a better linkage of clinical rotations with the theory being taught in the pre-clinical modules, and (ii) a more effective introduction to best practices for antimicrobial use in clinical practice. In this respect, international and national guidelines for antimicrobial use, which appear to be widely neglected in the current veterinary curricula, could provide advanced teaching on the clinical importance and use of different antimicrobial classes in the diverse animal species and disease conditions.

## Figures and Tables

**Table 1:** Variables with significant (*p* < 0.05) impact on the average perception of preparedness (APP) of European veterinary students in the fields of pharmacology of antimicrobial agents, clinical use of antimicrobial agents, and antimicrobial resistance (AMR) based on student-level mixed linear models; impact on students’ knowledge score (calculated as percentage of correct answers given to questions Q14–18, Q21–23 of the survey), based on student-level mixed linear models; and impact on sales of antimicrobial agents based on country-level linear models. Results are corrected for multiple comparisons by the Bonferroni–Holm method. Variables that were not included in one of these four models are indicated by ∄. Variables or values that were not significant in all four models are not included in the table.

Variable	Value			Impact on		
		APP Value in Pharmacology	APP Value in Clinical Use	APP Value in AMR	Knowledge Score	Sales of Antimicrobials
Grades	Better grades	Higher	Higher	Higher	Higher	∄
Clinical rotations	Completed	Not significant	Higher (compared to: “No, I will perform my clinical rotations later”)	∄	Higher (compared to: “No, I will perform my clinical rotations later”)	∄
Teaching by lectures	Higher frequency	Not significant	Higher	Higher	Not significant	∄
Teaching by discussions of clinical cases	Higher frequency	Not significant	Higher	Higher	∄	∄
Specialization	(See details for each model)	∄	Not significant	Lower in “Undecided” students compared to all other specializations	∄	∄
Satisfaction	Yes	Higher (compared to all other answers)	Higher (compared to all other answers)	Higher (compared to all other answers)	Higher (compared to “No, I feel I need more education on both general antibiotic treatment and rational antibiotic use” and “I don’t know”)	∄
Treatment of cystitis by systemic therapy	Higher % of students	∄	∄	∄	∄	Lower

## Data Availability

The data presented in this study are available on request from the corresponding author.

## Supplementary Materials

The following are available online at https://www.mdpi.com/article/10.3390/antibiotics10040364/s1, File S1: Contribution Log. Figure S1: Countries and veterinary schools included in the survey. Point size indicates the minimum number of complete answers included in the final analysis from each school, Figure S2: Weighted average perception of preparedness of European final-year veterinary students in topics related to pharmacology of antimicrobial agents (first column), clinical use of antimicrobial agents (second column), and antimicrobial resistance (third column), Figure S3: Perception of preparedness of European veterinary students in topics related to pharmacology of antimicrobial agents, clinical use of antimicrobial agents, and antimicrobial resistance (AMR). Values 4, 3, 2, 1, and 0, correspond to “Well prepared”, “Sufficiently prepared”, “Poorly prepared”, “Not at all prepared” and “I have not received any teaching/training in the topic”, respectively. Students are grouped based on question number 4 of the survey, “Have you already performed your clinical rotations?”. Total number of students within each group is displayed inside the boxes. Students are not represented in the box plots if they marked “I don’t know” in all the questions within a block, Figure S4: Most probable aetiology assigned by final-year veterinary students to four clinical cases presented to them in question 19 of the survey: “Which is the most common causative agent in the following infections?”. Bar plots show the percentage of students selecting each of the answers available (bottom legends). Maps display the percentage of students that selected the answer specified in the top-left legends. Bar plots may display added percentages above or below 100% due to rounding of the values, Figure S5: Relative contribution of veterinary use of antimicrobial agents to the clinical problems of resistant bacteria in humans according to final-year veterinary students in Europe, as reported in question 25 of the survey: “In your opinion what is the relative contribution of veterinary use of antimicrobials to the clinical problems of resistant bacteria in humans?”. The bar plot shows the percentage of students selecting each of the answers available (bottom legend). The map displays the average relative contribution per country, which was estimated assigning values to each answer: Very Low (0), Low (1), Medium (2), High (3), and Uncertain (NA). Bar plots may display added percentages above or below 100% due to rounding of the values, Table S1: List of Veterinary schools enrolled in the study and participation data, Table S2: List of fixed and random effects included in the final models fitted for data obtained from European final-year veterinary students related to antimicrobials in veterinary medicine, Table S3: Elements of education in antimicrobial stewardship in veterinary medicine, Table S4: Final version of the survey in English. Results are provided in bold for each question, taking into account 3423 complete answers collected in 11 available languages, Table S5: Statistical analyses performed on data generated in the survey.
