# Supplementary material for: Towards a Better and Harmonized Education in Antimicrobial Stewardship in European Veterinary Curricula
Source: Antibiotics (Basel). 2021 Mar 30;10(4):364. doi: 10.3390/antibiotics10040364 (PMC8067070; doi:10.3390/antibiotics10040364)
Supplement: Supplementary file 1 [file antibiotics-10-00364-s001.zip › Supplementary material Antibiotics.pdf]

SUPPLEMETERARY MATERIAL

1) **SUPPLEMETARY FIGURES**

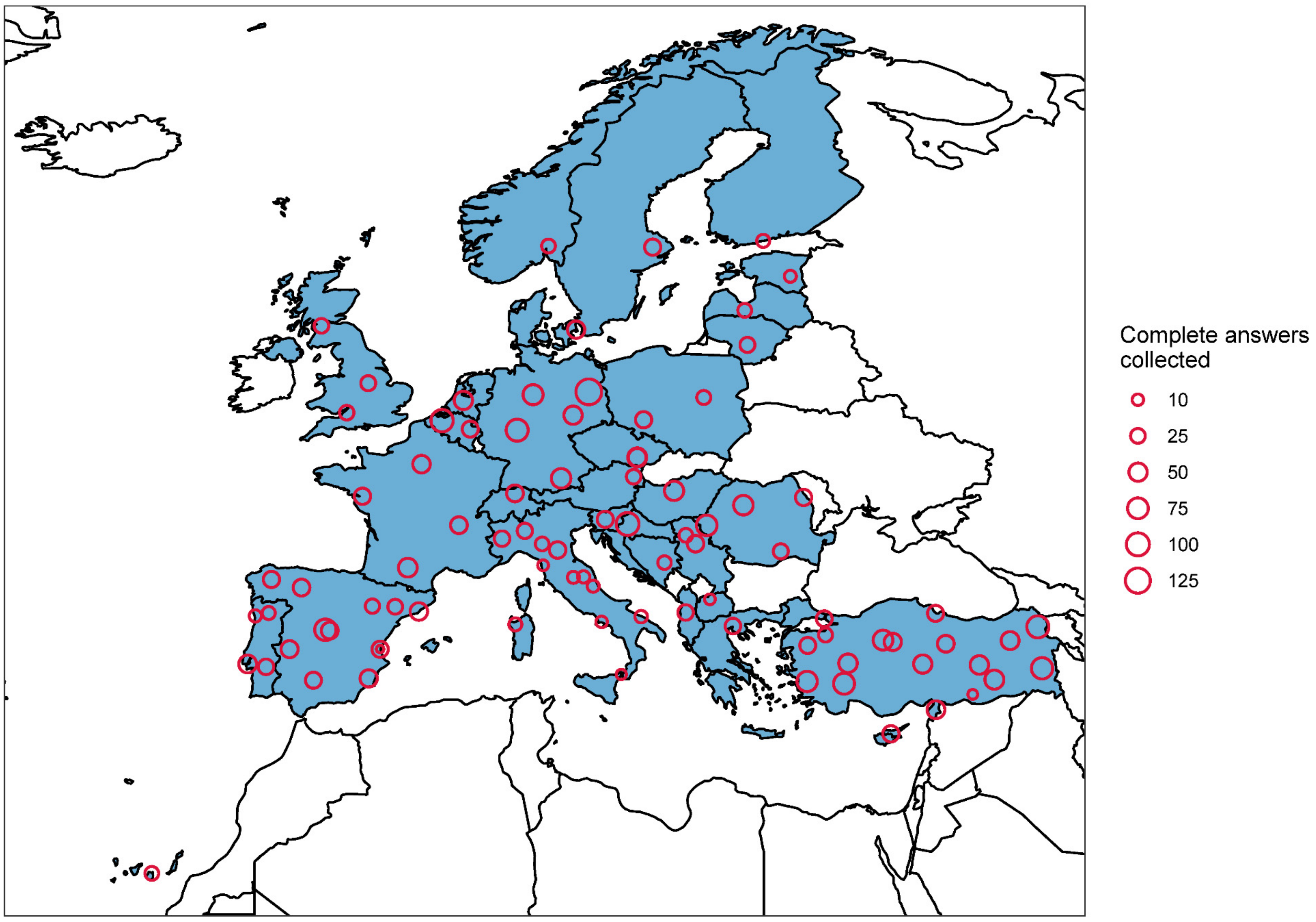

Figure S1. Countries and veterinary schools included in the survey. Point size indicates the minimum number of complete answers included in the final analysis from each school.

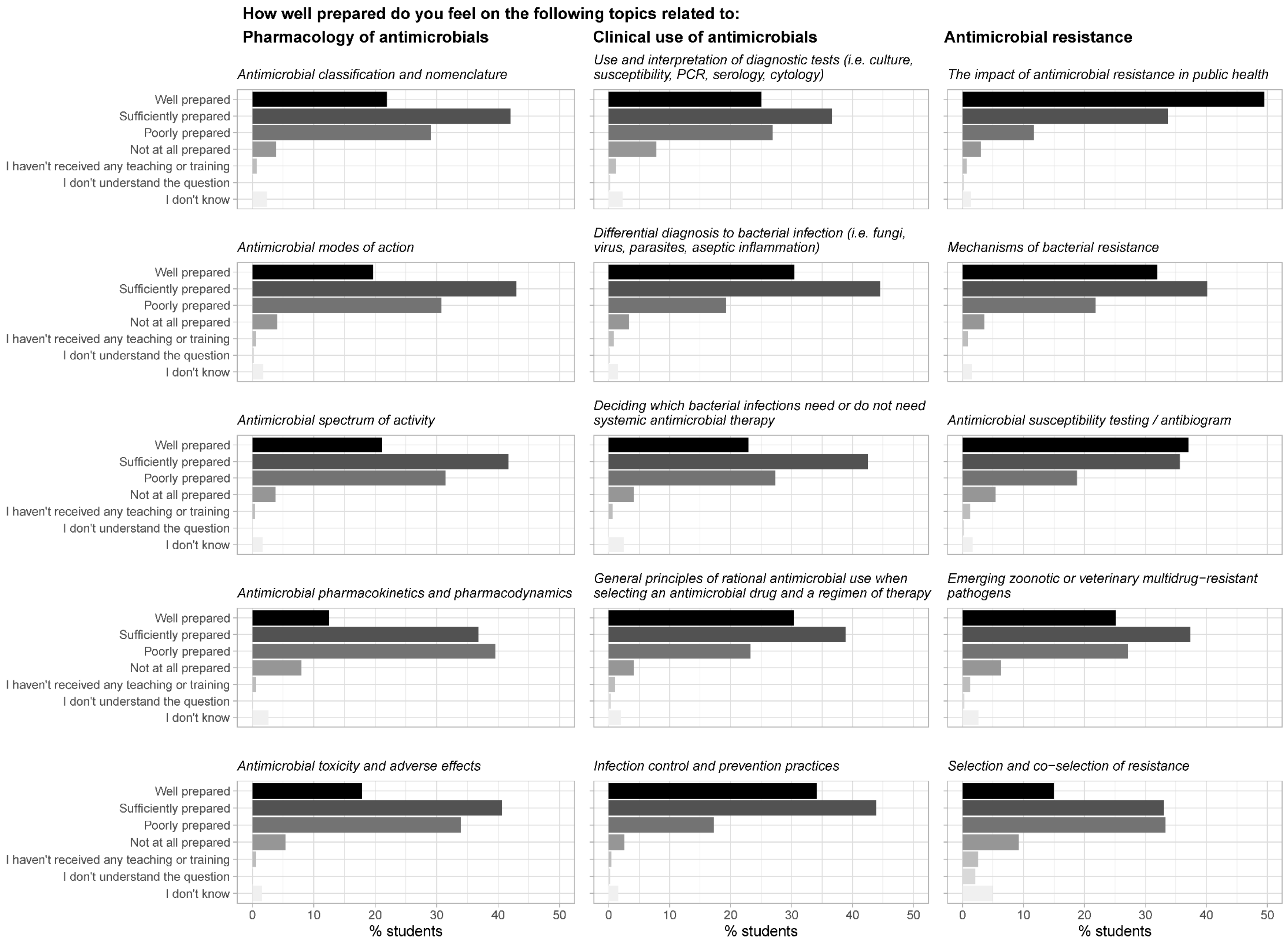

Figure S2. Weighted average perception of preparedness of European final-year veterinary students in topics related to pharmacology of antimicrobial agents (first column), clinical use of antimicrobial agents (second column), and antimicrobial resistance (third column).

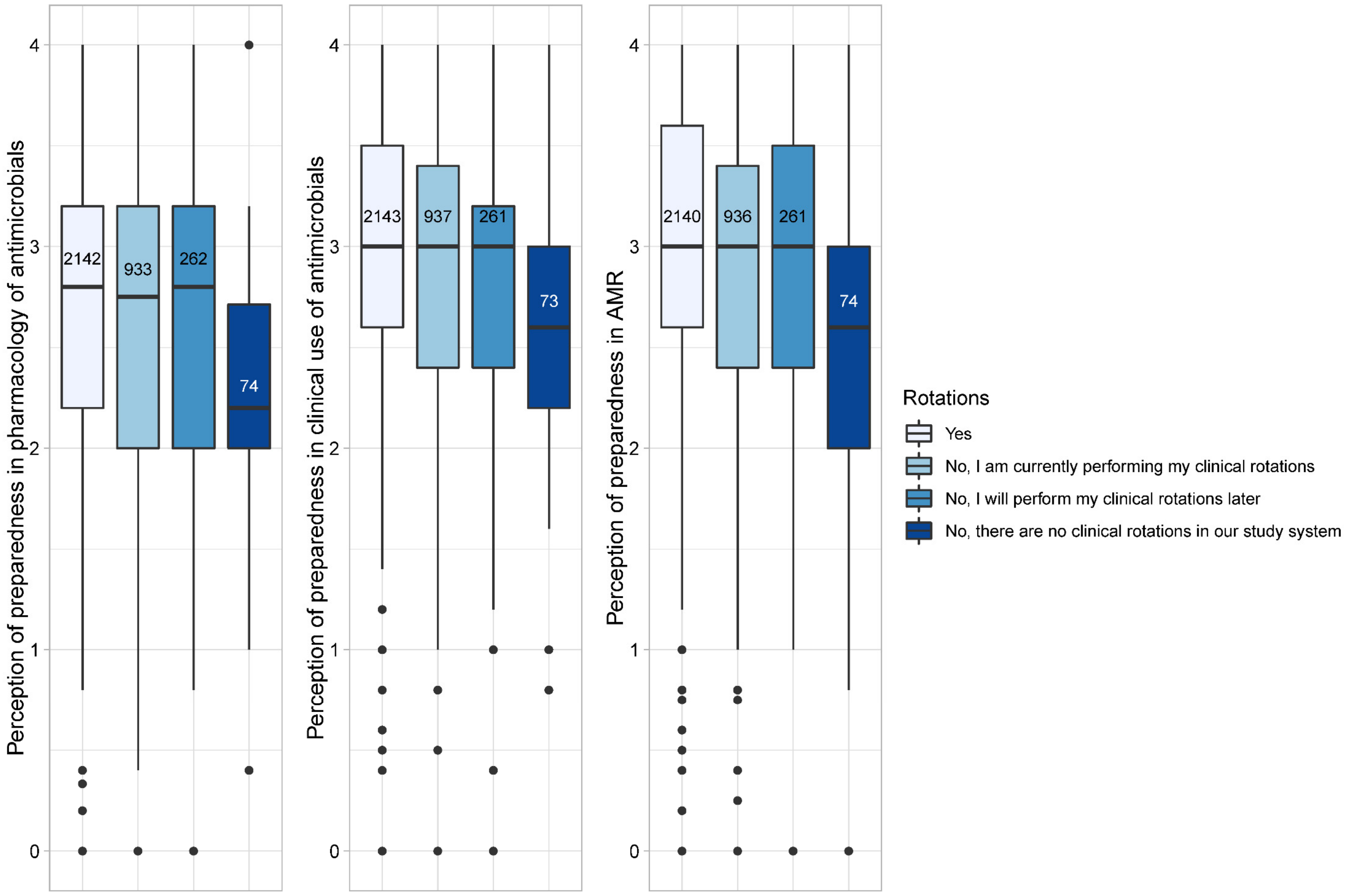

Figure S3. Perception of preparedness of European veterinary students in topics related to pharmacology of antimicrobial agents, clinical use of antimicrobial agents, and antimicrobial resistance (AMR). Values 4, 3, 2, 1, and 0, correspond to *Well prepared*, *Sufficiently prepared*, *Poorly prepared*, *Not at all prepared*, and *I have not received any teaching/training in the topic*, respectively. Students are grouped based on question number 4 of the survey, *Have you already performed your clinical rotations?* Total number of students within each group is displayed inside the boxes. Students are not represented in the box plots if they marked *I don't know* in all the questions within a block.

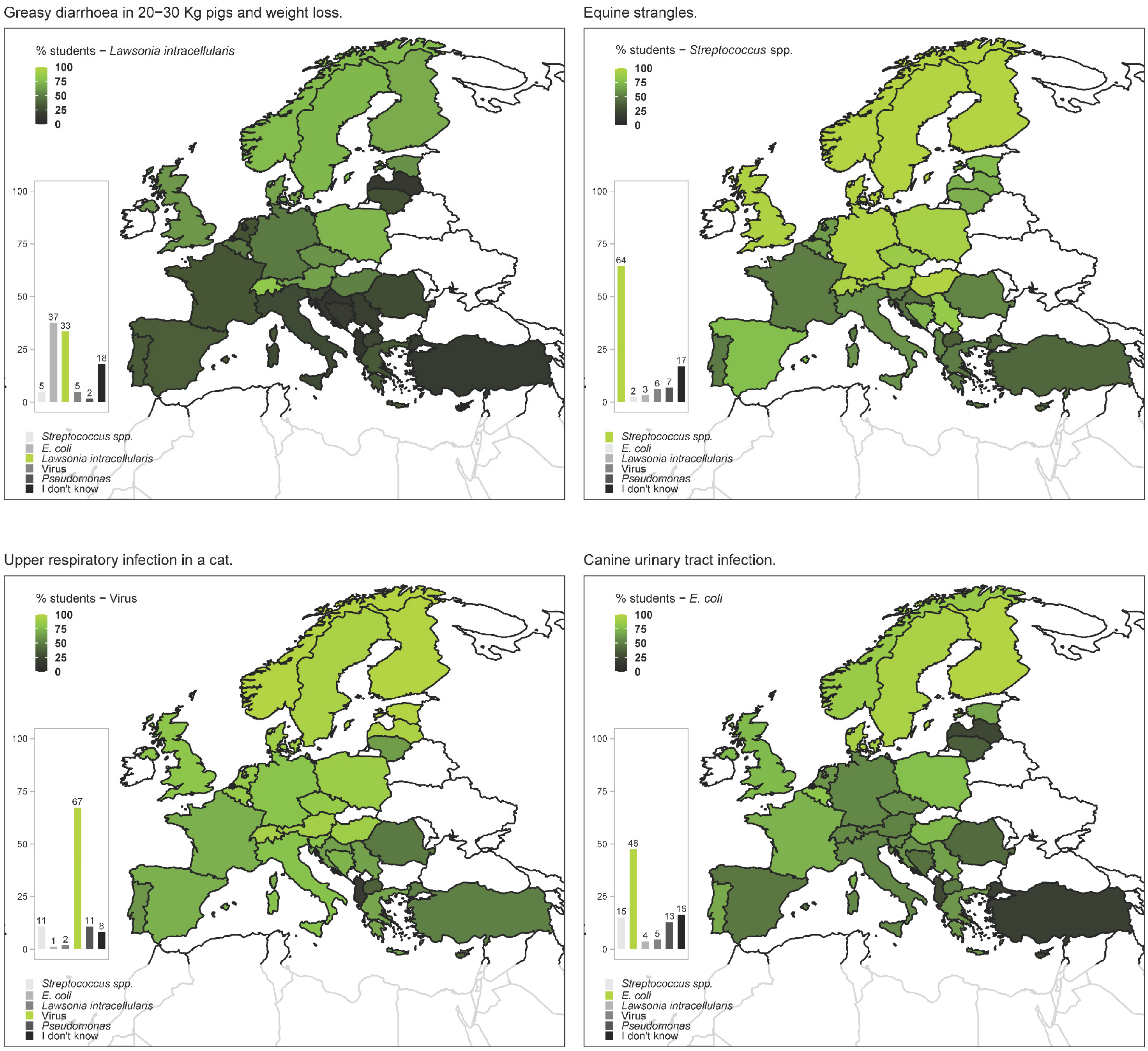

Figure S4. Most probable aetiology assigned by final-year veterinary students to four clinical cases presented to them in question 19 of the survey: *Which is the most common causative agent in the following infections?* Bar plots show the percentage of students selecting each of the answers available (bottom legends). Maps display the percentage of students that selected the answer specified in the top-left legends. Bar plots may display added percentages above or below 100 % due to rounding of the values.

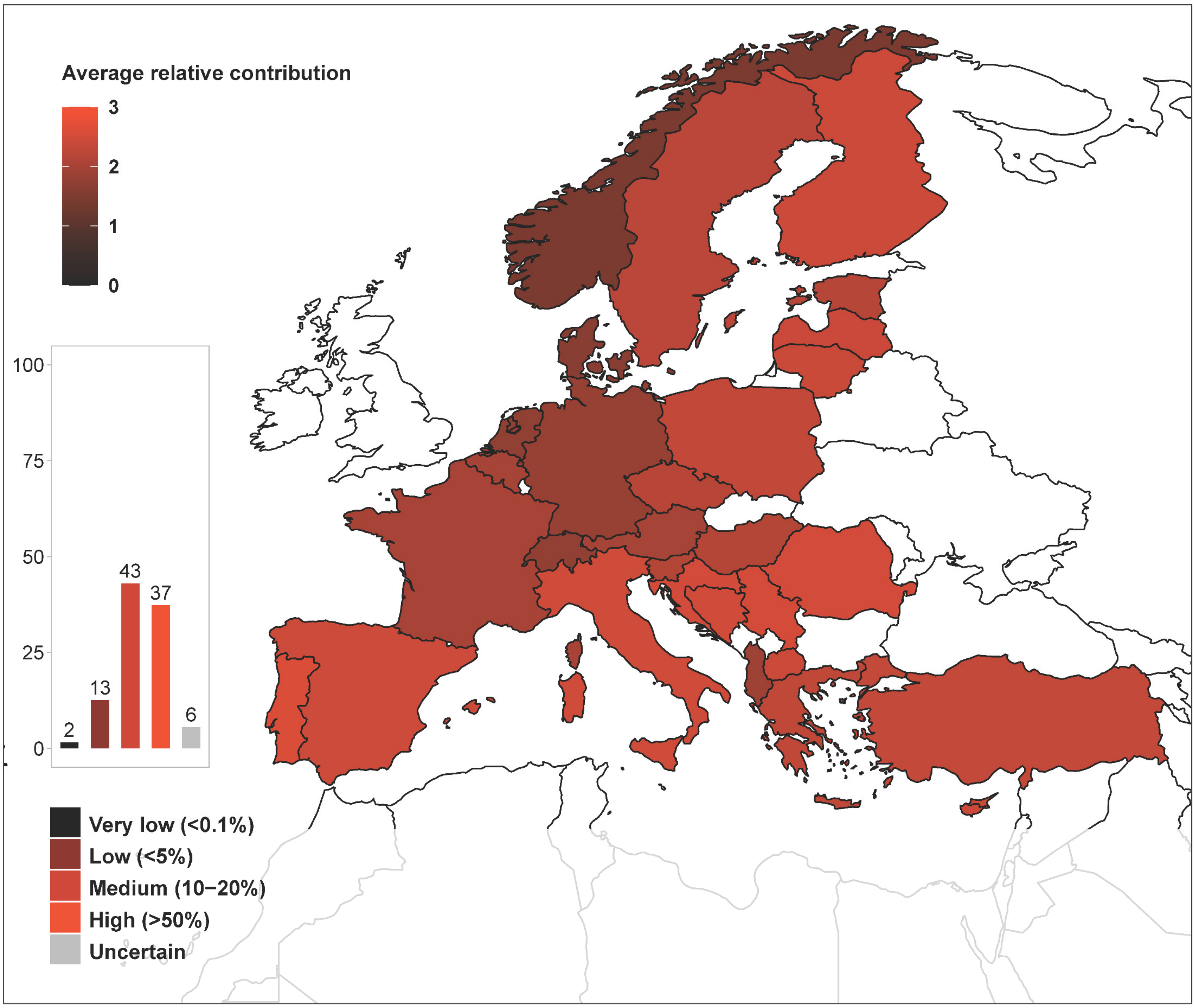

Figure S5. Relative contribution of veterinary use of antimicrobial agents to the clinical problems of resistant bacteria in humans according to final-year veterinary students in Europe, as reported in question 25 of the survey: *In your opinion what is the relative contribution of veterinary use of antimicrobials to the clinical problems of resistant bacteria in humans?* The bar plot shows the percentage of students selecting each of the answers available (bottom legend). The map displays the average relative contribution per country, which was estimated assigning values to each answer: *Very Low* (0), *Low* (1), *Medium* (2), *High* (3), and *Uncertain* (NA). Bar plots may display added percentages above or below 100 % due to rounding of the values.

2) SUPPLEMENTARY TABLES

Table S1. List of veterinary schools enrolled in the study and participation data.

Country	Veterinary school	Eligible students	Answers	Participation rate
Albania	Faculty of Veterinary Medicine, Agricultural University of Tirana	95	30	32%
Austria	University of Veterinary Medicine, Vienna	110	23	21%
Belgium	Faculty of Veterinary Medicine, Ghent University	200	91	46%
Belgium	Faculty of Veterinary Medicine, University of Liège	275	32	12%
Bosnia Herzegovina	University of Sarajevo, Veterinary faculty	31	18	58%
Croatia	Faculty of Veterinary Medicine, University of Zagreb	117	97	83%
Cyprus	Faculty of Veterinary Medicine, Near East University	34	34	100%
Czech	University of Veterinary and Pharmaceutical Sciences, Brno, Faculty of Veterinary Medicine/Czech and English programme	169	56	33%
Czech	University of Veterinary and Pharmaceutical Sciences, Brno, Faculty of Veterinary Hygiene and Ecology/Czech programme	80	46	58%
Denmark	Faculty of Health and Medical Sciences (SUND)	155	42	27%
Estonia	Estonian University of Life Sciences	45	12	27%
Finland	Faculty of Veterinary Medicine, University of Helsinki	66	16	24%
France	Veterinary School Alfort (EnvA)	130	44	34%
France	Veterinary School Lyon (VetAgro Sup)	135	38	28%
France	Oniris Nantes Veterinary School	132	35	27%
France	Veterinary School Toulouse	140	54	39%
Germany	Department of Veterinary Medicine, Freie Universität Berlin	320	136	43%
Germany	Justus, Liebig Universität Giessen	240	87	36%
Germany	University of Veterinary Medicine Hannover	281	67	24%
Germany	University of Leipzig	251	51	20%
Germany	Veterinary Faculty, LMU München	228	62	27%
Greece	Aristotle University of Thessaloniki Faculty of Veterinary Medicine	92	30	33%
Hungary	Állatorvostudományi Egyetem, University of Veterinary Medicine, Budapest	199	58	29%
Italy	Department of Veterinary Medicine, Bari	47	14	30%
Italy	Department of Medical Veterinary Sciences, Bolognia	80	37	46%
Italy	School of Biosciences and Veterinary Medicine, Camerino	23	13	57%
Italy	Department of Veterinary Sciences, Messina	18	7	39%
Italy	Department of Veterinary Medicine, Milano	92	28	30%
Italy	Department of Veterinary Medicine and Animal Production, Napoli	40	10	25%
Italy	Department of Medical Veterinary Sciences, Parma	45	19	42%
Italy	Department of Veterinary Medicine, Perugia	63	12	19%
Italy	Department of Veterinary Sciences, Pisa	42	8	19%
Italy	Department of Veterinary Medicine, Sassari	20	16	80%
Italy	Department of Clinical Veterinary Medicine, Teramo	19	12	63%
Italy	Department of Veterinary Sciences, Torino	89	30	34%
Latvia	Latvia University of Agriculture, Faculty of Veterinary Medicine	37	20	54%
Lithuania	Lithuanian University of Health Sciences, Kaunas	122	27	22%
Macedonia	Faculty of Veterinary Medicine, Skopje	17	8	47%
Netherlands	Faculty of Veterinary Medicine, Utrecht University	200	51	26%
Norway	Norwegian University of Life Science (NMBU), Oslo	60	20	33%
Poland	Warsaw University of Life Sciences, Warsaw	157	21	13%
Poland	Wrocław University of Environmental and Life Sciences	150	34	23%
Portugal	University of Evora	50	30	60%
Portugal	University of Lisbon	138	46	33%
Portugal	Instituto de Ciencias Biomedicas de Abel Salazar, Porto	90	13	14%
Portugal	Department of Veterinary Sciences, University of Trás os Montes and Alto Douro, Vila Real	75	16	21%
Romania	Faculty of Veterinary Medicine, University of Agricultural Sciences and Veterinary Medicine, Bucharest	201	26	13%
Romania	Faculty of Veterinary Medicine, University of Agricultural Sciences and Veterinary Medicine Cluj Napoca	197	61	31%
Romania	Faculty of Veterinary Medicine Iasi, The University of Agricultural Sciences and Veterinary Medicine Iaşi	105	35	33%
Romania	Faculty of Veterinary Medicine, Banat University of Agricultural Sciences and Veterinary Medicine Timisoara	113	72	64%
Serbia	Faculty of Veterinary Medicine, University of Belgrade	63	37	59%
Serbia	Faculty of Agriculture, Department for Veterinary medicine, University of Novi Sad	45	19	42%
Slovenia	University of Ljubljana, Veterinary Faculty	53	34	64%

Spain	Universidad Alfonso X el Sabio	103	81	79%
Spain	Universidad Autónoma de Barcelona	126	44	35%
Spain	Universidad Católica de Valencia San Vicente mártir	29	4	14%
Spain	Universidad CEU Cardenal Herrera	117	34	29%
Spain	Universidad Complutense de Madrid	155	38	25%
Spain	Universidad de Córdoba	125	33	26%
Spain	Universidad de Extremadura, Cáceres	116	40	34%
Spain	Universidad de las Palmas de Gran Canaria	95	20	21%
Spain	Universidad de León	106	38	36%
Spain	Universidad de Lerida	40	25	63%
Spain	Universidad de Murcia	98	41	42%
Spain	Universidad de Santiago de Compostela, Lugo	158	34	22%
Spain	Universidad de Zaragoza	167	22	13%
Sweden	Swedish University of Agricultural Sciences (SLU), Uppsala	80	34	43%
Switzerland	Vetsuisse, Faculty University of Berne and Zurich	110	38	35%
Turkey	Adnan Menderes University Faculty of Veterinary Medicine, Aydın	70	70	100%
Turkey	Afyon Kocatepe University Faculty of Veterinary Medicine, Afyonkarahisar	60	52	87%
Turkey	Ankara University Faculty of Veterinary Medicine, Ankara	90	65	72%
Turkey	Atatürk University Faculty of Veterinary Medicine, Erzurum	47	47	100%
Turkey	Balıkesir University Faculty of Veterinary Medicine, Balıkesir	35	33	94%
Turkey	Cumhuriyet University Faculty of Veterinary Medicine, Sivas	52	38	73%
Turkey	Dicle University Faculty of Veterinary Medicine, Diyarbakır	56	56	100%
Turkey	Erciyes University Faculty of Veterinary Medicine, Kayseri	51	51	100%
Turkey	Fırat University Faculty of Veterinary Medicine, Elazığ	60	53	88%
Turkey	Harran University Faculty of Veterinary Medicine, Şanlı Urfa	20	6	30%
Turkey	İstanbul University Faculty of Veterinary Medicine, İstanbul	145	31	21%
Turkey	Kafkas University Faculty of Veterinary Medicine, Kars	89	89	100%
Turkey	Kırıkkale University Faculty of Veterinary Medicine, Kırıkkale	60	41	68%
Turkey	Mehmet Akif Ersoy University Faculty of Veterinary Medicine, Burdur	76	76	100%
Turkey	Mustafa Kemal University Faculty of Veterinary Medicine, Hatay	46	46	100%
Turkey	Ondokuz Mayıs University Faculty of Veterinary Medicine, Samsun	59	33	56%
Turkey	Uludağ University Faculty of Veterinary Medicine, Bursa	80	21	26%
Turkey	Yüzüncü Yıl University Faculty of Veterinary Medicine, Van	88	80	91%
United Kingdom	Bristol School of Veterinary Sciences, University of Bristol	118	23	19%
United Kingdom	Glasgow School of Veterinary Medicine, University of Glasgow	122	24	20%
United Kingdom	Nottingham School of Veterinary Medicine and Science, University of Nottingham	121	27	22%

Table S2. List of fixed and random effects included in the final models fitted for data obtained from European final-year veterinary students related to antimicrobials in veterinary medicine.

Outcome	Random effect	Fixed effects included in the final model
Average perception of preparedness in pharmacology of antimicrobials	(1|country/school)	Age, Sex, Grades, Lectures, Case discussion, Assignments, E-Learning, Rotations, Satisfaction
Average perception of preparedness in clinical use of antimicrobials	(1|country/school)	Age, Grades, Specialisation, Lectures, Case discussion, Assignments, Rotations, Satisfaction
Average perception of preparedness in antimicrobial resistance	(1|country/school)	Grades, Specialisation, Lectures, Case discussion, Assignments, Satisfaction
Knowledge score	(1|country/school)	Sex, Grades, Lectures, Assignments, Rotations, Satisfaction
Overall antimicrobial sales	-	APP in AMR, Systemic therapy for cystitis, Antiseptic therapy for superficial pyoderma, Small group teaching, Assignments, No familiarity with any guideline, Satisfaction

Table S3. Elements of education in antimicrobial stewardship in veterinary medicine.

Topic	Concept	Field/discipline	Principles
**ANTIMICROBIAL RESISTANCE AS A SOCIETAL PROBLEM (perception of the problem)**			
Impact	Impact on public health	Microbiology, infectious diseases, veterinary public health	▪ Consequences on human mortality and morbidity
	Impact on economy	Microbiology, infectious diseases, veterinary public health	▪ Consequences on costs for national healthcare systems, farmers and pet owners
	Impact on animal health	Microbiology, infectious diseases, veterinary public health	▪ Consequences on animal mortality and morbidity
Veterinary implications	Zoonotic transmission	Microbiology, infectious diseases, epidemiology, veterinary public health, occupational health	▪ Foodborne transmission vs. transmission by direct contact with animals<br>▪ Causal link between antimicrobial agent consumption and antimicrobial resistance
**BASIC KNOWLEDGE OF ANTIMICROBIAL AGENTS AND ANTIMICROBIAL RESISTANCE (scientific knowledge)**			
Antimicrobial agents	Definition	Pharmacology, microbiology	▪ Antibiotic vs biocide (selective toxicity)
	Classification	Pharmacology, microbiology	▪ Classification of veterinary antimicrobial agents based on chemical structure<br>▪ Bactericidal vs. bacteriostatic drugs<br>▪ Time-dependent vs. concentration-dependent drugs
	Mechanism of action	Pharmacology, microbiology	▪ Main mechanisms of action of antimicrobial agents used in veterinary medicine (inhibition of cell wall synthesis, protein synthesis and DNA synthesis)
	Spectrum	Pharmacology, microbiology, infectious diseases, clinical medicine	▪ Broad vs. narrow spectrum antimicrobial agents, advantages and disadvantages<br>▪ Activity against main veterinary pathogens
	Toxicity	Toxicology, pharmacology, food safety, ecology	▪ Adverse reactions in different animal species<br>▪ Withdrawal times for different antimicrobial agents<br>▪ Consequences of antimicrobial residues in food<br>▪ Consequences of antimicrobial residues in the environment
	Use	Pharmacology, microbiology, clinical medicine, herd management	▪ Therapy vs. metaphylaxis/prophylaxis<br>▪ Dose, administration interval and treatment duration
Antimicrobial resistance	Definition	Microbiology, pharmacology	▪ Acquired vs intrinsic/natural resistance
	Selection	Microbiology, pharmacology	▪ Selection and co-selection by antibiotics and non-antibiotics (e.g. zinc)
	Acquisition	Microbiology, pharmacology	▪ Mutation vs horizontal gene transfer
	Measurement	Microbiology, pharmacology	▪ Definition of MIC/breakpoint<br>▪ Methods for susceptibility testing<br>▪ Antimicrobial agent selection based on MIC susceptibility results
	Mechanisms	Microbiology, pharmacology	▪ Main mechanisms of resistance (enzymatic inactivation, target modification, efflux, reduced permeability, etc.)
	Epidemiology	Microbiology, infectious diseases, veterinary public health	▪ Clonal spread vs horizontal gene transfer<br>▪ Mechanisms of horizontal gene transfer (conjugation, transformation, transduction)
	Resistant bacteria of veterinary interest	Microbiology, infectious diseases, clinical medicine, veterinary public health, zoonoses	▪ Antimicrobial resistance in zoonotic pathogens (e.g. Salmonella and Campylobacter)<br>▪ Emerging zoonotic threats (MRSA, MRSP, ESBL-producing and colistin-resistant E. coli)<br>▪ Antimicrobial resistance in veterinary pathogens (e.g. MRSA, MRSP, MDR E. coli)
**CONTROL OF ANTIMICROBIAL RESISTANCE (practical implications)**			
Rational antimicrobial use	Legal measures to control consumption	Veterinary public health, legal medicine	▪ Effects of legal measures (ban, restrictions, penalties, disincentives, etc)
	Correct diagnosis of bacterial infection	Immunology, clinical microbiology, infectious diseases, clinical medicine	▪ Recognition of clinical signs of infection and severity<br>▪ Interpretation of clinical and laboratory biological markers<br>▪ Differential diagnosis between bacterial and viral infections
	Correct use of the microbiology laboratory	Clinical microbiology, infectious diseases, clinical medicine	▪ Clinical situations where diagnostic microbiology is recommended<br>▪ Choice of specimen type for common infections<br>▪ Interpretation of culture and susceptibility test results
	Empiric therapy	Clinical microbiology, clinical medicine	▪ Infections for which therapy is not required (self-limiting infections)<br>▪ Infections for which topical therapy is sufficient<br>▪ Follow-up and re-assessment of empiric therapy on the basis of clinical outcome and culture results
	Rational antimicrobial choice	Pharmacology, clinical microbiology, infectious diseases, clinical medicine	▪ Choice based on knowledge of general and local resistance trends in the most likely target pathogen<br>▪ Choice based on antimicrobial activity at the infection site<br>▪ Choice based on PK/PD ratio<br>▪ Choice based on local/national/ international antimicrobial guidelines<br>▪ Choice guided by point-of-care tests (e.g. cytology and culture-based commercial tests)<br>▪ Choice based on severity of infection (life threatening Vs. chronic/non-systemic)<br>▪ Benefits of combination therapy
	Rational dosage regimens	Pharmacology, infectious diseases, clinical medicine	▪ Choice of administration route and treatment regimen (dose, interval, duration) based on (1) infection site, (2) acute/chronic, (3) toxicity.<br>▪ Effects of dose, administration interval and treatment duration on selection of resistance
	Prescription practices	Communication, psychology	▪ Justification to animal owners/farmers for non-antimicrobial prescription<br>▪ Education of owners/farmers to compliance

Prevention of transmission	Hospital infection control	Clinical microbiology, infectious diseases, surgery	▪ Best practices for hospital infection control<br>▪ Management of patients infected with MDR bacteria<br>▪ Advise owners for management of pets colonized/infected with MDR bacteria
	Farm biosecurity	Clinical medicine, herd management, veterinary public health	▪ Measure to prevent transmission between and within herds
	Food hygiene	Food safety, veterinary public health	▪ Best slaughterhouse and kitchen hygiene practices
Pharmacovigilance	Pharmacology	Human and animal health	▪ Report on decrease/lack of efficacy<br>▪ Report of notifiable diseases

Table S4. Final version of the survey in English. Results are provided in bold for each question, taking into account 3423 complete answers collected in 10 languages.

Questions about the student	
Q1. Which year were you born?	
(Drop-down options provided)	**Min. 1964**<br>**Median 1992**<br>**Mean 1993**<br>**Max. 2000.**
Q2. What is you gender?	
Female	**65.3%**
Male	**34.7%**
Q3. Are you in the final year (or final two semesters) to become a veterinarian?	
Yes	**100%**
No	**0%**
Q4. Have you already performed clinical rotations?	
Yes	**62.7%**
No, I am currently performing my clinical rotations	**27.4%**
No, I will perform my clinical rotations later	**7.7%**
No, there are no clinical rotations in our study system	**2.2%**
Q5. Will you have the possibility to prescribe antimicrobials after this year (or the final two semesters)?	
Yes	**92.6%**
No. I have to do an specialization / internship after this year before I can prescribe antimicrobials	**7.4%**
No, because this is not my final year to become a veterinarian	**0%**
Q6. Which veterinary school are you studying at?*	
Albania. Faculty of Veterinary Medicine. Agricultural University of Tirana	**0.9%**
Austria. University of Veterinary Medicine, Vienna	**0.7%**
Belgium. Faculty of Veterinary Medicine. Ghent University	**2.7%**
Belgium. Faculty of Veterinary Medicine. University of Liège	**0.9%**
Bosnia Herzegovina. University of Sarajevo. Veterinary faculty	**0.5%**
Croatia. Faculty of Veterinary Medicine. University of Zagreb	**2.8%**
Cyprus. Faculty of Veterinary Medicine. Near East University	**1.0%**
Czech. University of Veterinary and Pharmaceutical Sciences, Brno, Faculty of Veterinary Hygiene and Ecology/Czech programme	**1.3%**
Czech. University of Veterinary and Pharmaceutical Sciences, Brno, Faculty of Veterinary Medicine/Czech and English programme	**1.6%**
Denmark. Faculty of Health and Medical Sciences. SUND	**1.2%**
Estonia. Estonian University of Life Sciences	**0.4%**
Finland. Faculty of Veterinary Medicine. University of Helsinki	**0.5%**
France. Oniris Nantes Veterinary School	**1.0%**
France. Veterinary School Alfort. EnvA	**1.3%**
France. Veterinary School Lyon. VetAgro Sup	**1.1%**
France. Veterinary School Toulouse	**1.6%**
Germany. Department of Veterinary Medicine. Freie Universität Berlin	**4.0%**
Germany. Justus-Liebig Universität Giessen	**2.5%**
Germany. University of Leipzig	**1.5%**
Germany. University of Veterinary Medicine Hannover	**2.0%**
Germany. Veterinary Faculty. LMU München	**1.8%**
Greece. Aristotle University of Thessaloniki Faculty of Veterinary Medicine	**0.9%**
Hungary. Állatorvostudományi Egyetem. Budapest. University of Veterinary Medicine	**1.7%**
Italy. Department of Clinical Veterinary Medicine. Teramo	**0.4%**
Italy. Department of Medical-Veterinary Sciences. Parma	**0.6%**
Italy. Department of Medical Veterinary Sciences. Bolognia	**1.1%**
Italy. Department of Veterinary Medicine and Animal Production. Napoli	**0.3%**
Italy. Department of Veterinary Medicine. Bari	**0.4%**
Italy. Department of Veterinary Medicine. Milano	**0.8%**
Italy. Department of Veterinary Medicine. Perugia	**0.4%**
Italy. Department of Veterinary Medicine. Sassari	**0.5%**
Italy. Department of Veterinary Sciences. Pisa	**0.2%**
Italy. Department of Veterinary Sciences. Messina	**0.2%**
Italy. Department of Veterinary Sciences. Torino	**0.9%**
Italy. School of Biosciences and Veterinary Medicine. Camerino	**0.4%**
Latvia. Latvia University of Agriculture. Faculty of Veterinary Medicine	**0.6%**
Lithuania. Lithuanian University of Health Sciences. Kaunas	**0.8%**
Macedonia. Faculty of Veterinary Medicine. Skopje	**0.2%**
Netherlands. Faculty of Veterinary Medicine. Utrecht University	**1.5%**
Norway. Norwegian University of Life Science. NMBU-Oslo	**0.6%**
Poland. Warsaw University of Life Sciences. Warsaw	**0.6%**
Poland. Wrocław University of Environmental and Life Sciences	**1.0%**
Portugal. Department of Veterinary Sciences, University of Trás-os-Montes and Alto Douro, Vila Real	**0.5%**
Portugal. Instituto de Ciencias Biomedicas de Abel Salazar. Porto	**0.4%**

Portugal. University of Evora	**0.9%**
Portugal. University of Lisbon	**1.3%**
Romania. Faculty of Veterinary Medicine Iasi. The University of Agricultural Sciences and Veterinary Medicine Iaşi	**1.0%**
Romania. Faculty of Veterinary Medicine. University of Agricultural Sciences and Veterinary Medicine Cluj Napoca	**1.8%**
Romania. Faculty of Veterinary Medicine. University of Agricultural Sciences and Veterinary Medicine. Bucharest	**0.8%**
Romania. Faculty of Veterinary Medicine. Banat University of Agricultural Sciences and Veterinary Medicine Timisoara	**2.1%**
Serbia. Faculty of Agriculture. Department for Veterinary medicine, University of Novi Sad	**0.6%**
Serbia. Faculty of Veterinary Medicine. University of Belgrade	**1.1%**
Slovenia. University of Ljubljana, Veterinary Faculty	**1.0%**
Spain. Universidad Alfonso X el Sabio	**2.4%**
Spain. Universidad Autónoma de Barcelona	**1.3%**
Spain. Universidad Católica de Valencia San Vicente mártir	**0.1%**
Spain. Universidad CEU Cardenal Herrera	**1.0%**
Spain. Universidad Complutense de Madrid	**1.1%**
Spain. Universidad de Córdoba	**1.0%**
Spain. Universidad de Extremadura. Cáceres	**1.2%**
Spain. Universidad de las Palmas de Gran Canaria	**0.6%**
Spain. Universidad de León	**1.1%**
Spain. Universidad de Lerida	**0.7%**
Spain. Universidad de Murcia	**1.2%**
Spain. Universidad de Santiago de Compostela. Lugo	**1.0%**
Spain. Universidad de Zaragoza	**0.6%**
Sweden. Swedish University of Agricultural Sciences. SLU. Uppsala	**1.0%**
Switzerland. Vetsuisse-Faculty University of Berne and Zurich	**1.1%**
Turkey. Adnan Menderes University Faculty of Veterinary Medicine, Aydın	**2.0%**
Turkey. Afyon Kocatepe University Faculty of Veterinary Medicine, Afyonkarahisar	**1.5%**
Turkey. Ankara University Faculty of Veterinary Medicine, Ankara	**1.9%**
Turkey. Atatürk University Faculty of Veterinary Medicine, Erzurum	**1.4%**
Turkey. Balıkesir University Faculty of Veterinary Medicine, Balıkesir	**1.0%**
Turkey. Cumhuriyet University Faculty of Veterinary Medicine, Sivas	**1.1%**
Turkey. Dicle University Faculty of Veterinary Medicine, Diyarbakır	**1.6%**
Turkey. Erciyes University Faculty of Veterinary Medicine, Kayseri	**1.5%**
Turkey. Fırat University Faculty of Veterinary Medicine, Elazığ	**1.5%**
Turkey. Harran University Faculty of Veterinary Medicine, Şanlı Urfa	**0.2%**
Turkey. İstanbul University Faculty of Veterinary Medicine, İstanbul	**0.9%**
Turkey. Kafkas University Faculty of Veterinary Medicine, Kars	**2.6%**
Turkey. Kırıkkale University Faculty of Veterinary Medicine, Kırıkkale	**1.2%**
Turkey. Mehmet Akif Ersoy University Faculty of Veterinary Medicine, Burdur	**2.2%**
Turkey. Mustafa Kemal University Faculty of Veterinary Medicine, Hatay	**1.3%**
Turkey. Ondokuz Mayıs University Faculty of Veterinary Medicine, Samsun	**1.0%**
Turkey. Uludağ University Faculty of Veterinary Medicine, Bursa	**0.6%**
Turkey. Yüzüncü Yıl University Faculty of Veterinary Medicine, Van	**2.3%**
United Kingdom. Bristol School of Veterinary Sciences- University of Bristol	**0.7%**
United Kingdom. Glasgow School of Veterinary Medicine. University of Glasgow	**0.7%**
United Kingdom. Nottingham School of Veterinary Medicine and Science. University of Nottingham	**0.8%**
Q7. What is the minimum duration of the education to become a veterinarian (DVM) in your country?*	
4 years	**0.3%**
5 years	**53.4%**
5.5 years	**20.3%**
6 years	**23%**
6.5 years	**0.1%**
7 years	**2.3%**
Q8. Are you a citizen in the country where you study veterinary medicine?	
Yes	**98.8%**
No	**7.2%**
Q9. In a scale from 1 to 10, (10 representing a top student, 5 an average student, and 1 a student at the bottom of the rank), how would you rank your overall performance as a student compared to your classmates (during your entire education as a veterinarian)?	
10	**3.6%**
9	**8.2%**
8	**25.5%**
7	**28.6%**
6	**16.9%**
5	**12.6%**
4	**2.7%**
3	**1.1%**
2	**0.4%**
1	**0.3%**
Q10. In what kind of field would you like to work?	

Small / companion animal clinic or equine clinic	45.5%
Food animal practitioner (e.g. cattle, pigs, poultry, small ruminants, fish)	27.4%
Other	13.8%
Not decided	13.3%

Evaluation of the curricula. Students' perception of preparedness.

Q11. How well prepared do you feel on the following topics related to pharmacology of antimicrobials?

	I haven't received any teaching / training in this topic	Not at all prepared	Poorly prepared	Sufficiently prepared	Well prepared	I don't know	I don't understand the question
Antimicrobial classification and nomenclature	0.7%	3.9%	29.1%	42%	21.8%	2.4%	0.1%
Antimicrobial modes of action	0.6%	4%	30.8%	43%	19.6%	1.8%	0.2%
Antimicrobial spectrum of activity	0.4%	3.7%	31.4%	41.6%	21.1%	1.6%	0.1%
Antimicrobial pharmacokinetics and pharmacodynamics	0.6%	7.9%	39.6%	36.8%	12.4%	2.6%	0.1%
Antimicrobial toxicity and adverse effects	0.6%	5.4%	33.9%	40.7%	17.8%	1.5%	0.1%

Q12. How well prepared do you feel on the following topics related to clinical use of antimicrobials?

	I haven't received any teaching / training in this topic	Not at all prepared	Poorly prepared	Sufficiently prepared	Well prepared	I don't know	I don't understand the question
Use and interpretation of diagnostic tests (i.e. culture, susceptibility, PCR, serology, cytology)	1.2%	7.8%	26.9%	36.7%	25%	2.2%	0.2%
Differential diagnosis to bacterial infection (i.e. fungi, virus, parasites, aseptic inflammation)	0.8%	3.4%	19.2%	44.6%	30.4%	1.5%	0.1%
Deciding which bacterial infections need or do not need systemic antimicrobial therapy	0.6%	4.1%	27.3%	42.5%	22.9%	2.5%	0.1%
General principles of rational antimicrobial use when selecting an antimicrobial drug and a regimen of therapy	1%	4.1%	23.3%	38.9%	30.4%	2%	0.4%
Infection control and prevention practices	0.4%	2.6%	17.2%	43.9%	34.1%	1.5%	0.2%

Q13. How well prepared do you feel on the following topics related to antimicrobial resistance?

	I haven't received any teaching / training in this topic	Not at all prepared	Poorly prepared	Sufficiently prepared	Well prepared	I don't know	I don't understand the question
The impact of antimicrobial resistance in public health	0.6%	3%	11.7%	33.7%	49.4%	1.3%	0.2%
Mechanisms of bacterial resistance	0.9%	3.6%	21.8%	40.1%	31.9%	1.5%	0.1%
Antimicrobial susceptibility testing / antibiogram	1.3%	5.3%	18.8%	35.7%	37%	1.7%	0.2%
Emerging zoonotic or veterinary multidrug-resistant pathogens	1.3%	6.3%	27%	37.4%	25.2%	2.6%	0.3%
Selection and co-selection of resistance	2.5%	9.2%	33.3%	33%	15%	5%	2%

Practical questions

Q14. Which of the following antimicrobials is a lincosamide?

Ceftiofur	5.1%
Clindamycin	63%
Enrofloxacin	3.4%
Gentamicin	4.4%
Oxytetracycline	1.4%
I don't know	22.8%

Q15. Which of the following β-lactam antimicrobials has the broadest spectrum?

Ampicillin	4%
Amoxicillin	8.2%
Amoxicillin-clavulanic acid	64.1%
Penicillin	18.6%
I don't know	5.2%

Q16. Which of the following antimicrobials can cause kidney damage?

Clindamycin	5%
Doxycycline	15.5%
Erythromycin	11.1%
Gentamicin	50%
Penicillin	3.8%
I don't know	14.6%

Q17. What is an extended-spectrum β-lactamase (ESBL)?	AT, CH, DE †	Other countries
A host enzyme reducing in vivo activity of β-lactam antimicrobials	-	10.1%
† A bacterial enzyme reducing in vivo activity of β-lactam antimicrobials	36.9%	-
A β-lactam antimicrobial with broad spectrum	5.6%	12%
A bacterial enzyme hydrolysing β-lactam antimicrobials	47%	42%

A yeast enzyme catalysing biosynthesis of extended-spectrum β-lactams	1.5%	8.7%
I don't know	9.1%	27.2%

Q18. Which of the following antimicrobials is used for laboratory detection of methicillin resistance in staphylococci?	
Amoxicillin	10.6%
Methicillin	31.3%
Metronidazole	5.5%
Oxacillin	12.1%
I don't know	40.5%

Q19. Which is the most common causative agent in the following infections?						
	Streptococcus spp.	*E. coli*	*Lawsonia intracellularis*	Virus	*Pseudomonas*	I don't know
Greasy diarrhoea in 20-30 Kg pigs and weight loss	4.8%	37.5%	33.3%	4.8%	1.5%	18%
Equine strangles	64.4%	2.5%	3.2%	6.1%	6.7%	17%
Upper respiratory infection in cat	10.6%	1.3%	1.9%	67.3%	10.7%	8.1%
Canine urinary tract infection	15.1%	47.6%	3.7%	4.5%	12.7%	16.3%

Q20. Please indicate which treatment strategy you were taught to apply for the following infections:						
	Systemic antimicrobial therapy	Local antimicrobial therapy	Local antiseptic therapy	A combination of local and systemic	No treatment	I don't know
Cystitis	67.3%	8%	3.3%	14.8%	2.3%	4.4%
Subclinical bacteriuria	36.2%	7.1%	6.8%	7.4%	27.4%	15.2%
Canine superficial pyoderma	7.6%	31.5%	30.4%	22.2%	1.2%	7.1%
Severe bovine clinical mastitis	15.1%	16.4%	1.7%	60.9%	1.6%	4.3%

Q21. Which of the following strategies is NOT in line with the concept of antimicrobial stewardship?	
To maximise use of topical therapy for management of skin infections	22.4%
To make the best use of culture and susceptibility testing	4.8%
To minimise the use of antimicrobials that are critically important in human medicine	6.9%
To administer/prescribe antimicrobials at the lowest dose recommended by the manufacturer	28.2%
To administer / prescribe the shortest possible duration of antimicrobial therapy	22.1%
I don't know	15.7%
**Q22. Which of the following antimicrobial classes should be regarded as a second-line drug and reserved for management of complicated infections?**	
Aminopenicillins (e.g. amoxicillin)	6%
First-generation cephalosporins (e.g. cephalexin)	19.2%
Fluoroquinolones (e.g. enrofloxacin)	45.5%
Macrolides (e.g. erythromycin)	16.7%
I don't know	12.5%
**Q23. When using gloves, when should you wash your hands?**	
Before putting on the gloves	9.4%
After taking off the gloves	12.8%
Before and after using gloves	71.7%
There is no need to wash your hands if you are using gloves	3.7%
I don't know	2.4%
**Q24. Are you familiar with any practice guidelines for rational antimicrobial use? ‡**	
National (please specify which in the text box below)	26%
International (please specify which in the text box below)	6.2%
No	70.4%
**Opinion question**	
**Q25. In your opinion what is the relative contribution of veterinary use of antimicrobials to the clinical problems of resistant bacteria in humans?**	
High (>50%)	37.3%
Medium (10-20%)	43%
Low (<5%)	12.6%
Very low (<0.1%)	1.6%
Uncertain	5.6%

Question about teaching methods					
Q26. How often the following methods have been used to teach you on antimicrobials, antimicrobial resistance and antimicrobial use?					
	Very often	Sometimes	Rarely	Never	I don't know
Lectures	41.7%	37.9%	16.3%	1.8%	2.2%
Small group teaching	9.7%	33.9%	31.5%	22.7%	2.3%
Discussions of clinical cases	16.1%	40.6%	30.7%	10.9%	1.7%
Active learning assignment (article review, oral presentation)	9.3%	30.7%	32.8%	25%	2.2%
E-learning	5.8%	19.7%	28%	40.5%	6%
Clinical rotations	19.3%	40.4%	26.9%	9.7%	3.7%

Overall satisfaction with knowledge	
Q27. Overall do you think you receive adequate teaching to face antimicrobial and resistance issues in clinical practice?	
Yes	23.8%
No, I feel I had enough teaching on general antimicrobial treatment, but I need more on rational antimicrobial use	35.6%
No, I feel I need more education on both general antimicrobial treatment and rational antimicrobial use	37.1%
I don't know	3.4%

† Results of question 17 in Austria, Switzerland and Germany are reported separately from the rest of the countries due to an error in the translation of the survey to German.

‡ Question 24 allowed multiple answers. Sixty-one students reported both, national and international guidelines; 22 students reported both national and no guidelines; 8 students reported both international and no guidelines; one student reported all three options.

Table S5. Statistical analyses performed on data generated in the survey.

Point investigated	Methods
1) What is the impact of clinical rotations on students' perception of preparedness in each of the knowledge fields included in the survey (pharmacology, clinical use of antimicrobial agents, AMR)?	Pairwise Wilcoxon tests comparing perception of preparedness values between each of the stages of clinical rotations (i.e. *already completed the rotations*, *currently doing rotations*, *will do rotations later*, *no rotations in the curriculum*) using the stats package in R [1].
2) Which other factors have an impact on perception of preparedness?	Linear mixed models with outcome average perception of preparedness of each field were fitted with the lmerTest package in R [2,3]. Random effect (1 | country/school) was included to control for student, school and country. Model output was corrected for multiple comparisons by the Bonferroni-Holm method using the multcomp package in R [4].
3) Which factors have an impact on actual theoretical knowledge? (Perception of preparedness variables were excluded from this model. Correlation between knowledge and perception of preparedness was studied separately).	Linear mixed models with outcome theoretical knowledge (calculated from the block of practical questions) were fitted with the lmerTest package in R [2,3]. Random effect (1 | country/school) was included to control for student, school and country. Model output was corrected for multiple comparisons by the Bonferroni-Holm method using the multcomp package in R [4].
4) Associations between sales of antimicrobial agents for veterinary use in each country, with data from our survey.	Correlation and linear regression at the country level (dataset aggregated by country) using the stats package in R [1]. Model output was corrected for multiple comparisons by the Bonferroni-Holm method using the multcomp package in R [4].

References to methods in Table S5

1. R Core Team *R: A language and environment for statistical computing*, R Foundation for Statistical Computing: Vienna, Austria, 2018.
2. Bates, D.; Maechler, M.; Bolker, B.; Walker, S. Fitting Linear Mixed-Effects Models Using lme4. *Journal of Statistical Software* **2015**, *67*, 48, doi:doi:10.18637/jss.v067.i01.
3. Kuznetsova, A.; Brockhoff, P.B.; Christensen, R.H.B. lmerTest Package: Tests in Linear Mixed Effects Models. *2017* **2017**, *82*, 26, doi:10.18637/jss.v082.i13.
4. Hothorn, T.; Bretz, F.; Westfall, P. Simultaneous inference in general parametric models. *Biom J* **2008**, *50*, 346-363, doi:10.1002/bimj.200810425.
