# Supplementary material for: Towards a Better and Harmonized Education in Antimicrobial Stewardship in European Veterinary Curricula
Source: Antibiotics (Basel). 2021 Mar 30;10(4):364. doi: 10.3390/antibiotics10040364 (PMC8067070; doi:10.3390/antibiotics10040364)
Supplement: Supplementary file 1 [file antibiotics-10-00364-s001.zip › File S1-contribution log.ceg.pdf]

File S1. Contribution log

Collaborators list:

Naim Deniz Ayaz, Department of Food Hygiene and Technology, Faculty of Veterinary Medicine, Kirikkale University, Kirikkale 71450 Turkey; naimdenizayaz@kku.edu.tr

Ane Mohn Bjelland, Department of Paraclinical Sciences, Faculty of Veterinary Medicine, Norwegian University of Life Sciences, 1432 Ås, Norway; ane.mohn.bjelland@nmbu.no

Dorota Chrobak, Department of Pre-clinical Sciences, Faculty of Veterinary Medicine, Warsaw University of Life Sciences—SGGW, 02-786 Warsaw, Poland; Dorota.chrobak@wp.pl

Sonja Chvala-Mannsberger, Ethics and Animal Welfare Committee, Rectorate, University of Veterinary Medicine Vienna, 1210 Vienna, Austria; sonja.chvala@vetmeduni.ac.at

Jeroen Dewulf, Department of Reproduction, Obstetrics and Herd Health, Faculty of Veterinary Medicine, Ghent University, 8720 Merelbeke, Belgium; jeroen.dewulf@ugent.be

Monika Dolejská, Department of Biology and Wildlife Diseases, Faculty of Veterinary Hygiene and Ecology, University of Veterinary and Pharmaceutical Sciences Brno, 612 42 Brno, Czech Republic; monika.dolejska@gmail.com

Nihad Fejzic, Department of Epidemiology, Veterinary Faculty, University of Sarajevo, 71000 Sarajevo, Bosnia and Herzegovina; nihad.fejzic@vfs.unsa.ba

***George Filioussis**, Faculty of Veterinary Medicine, Aristotle University of Thessaloniki, 54124 Thessaloniki, Greece

László Fodor, Department of Microbiology and Infectious Diseases, University of Veterinary Medicine Budapest, 1143 Budapest, Hungary; fodor.laszlo@univet.hu

Savas Volkan Genc, Department of Veterinary Medicine History and Deontology, Faculty of Veterinary Medicine, Mehmet Akif Ersoy University, 15100 Burdur, Turkey; Department of Veterinary Medicine History and Deontology, Faculty of Veterinary Medicine, Near East University, 99150 Nicosia, Cyprus; svgenc@mehmetakif.edu.tr

Muammer Goncuoglu, Department of Food Hygiene and Technology, Faculty of Veterinary Medicine, Ankara University, 06110 Ankara, Turkey; muammer.goncuoglu@veterinary.ankara.edu.tr

Annamari Heikinheimo, Food Hygiene and Environmental Health Faculty of Veterinary Medicine, University of Helsinki, 00014 Helsinki, Finland; Finnish Food Authority, 60100 Seinäjoki, Finland; annamari.heikinheimo@helsinki.fi

Silvestra Kobal, Institute of Physiology, Pharmacology and Toxicology, Veterinary Faculty, University of Ljubljana, SI-1000 Ljubljana, Slovenia; silvestra.kobal@vf.uni-lj.si

Susanna Sternberg-Lewerin, Department of Biomedical Sciences and Veterinary Public Health, Swedish University of Agricultural Science, 750 07 Uppsala, Sweden; susanna.sternberg-lewerin@slu.se

Jani Mavromati, Department of Veterinary Public Health. Agricultural University of Tirana, 1025 Tirana, Albania; jmavromati@ubt.edu.al

Dušan Mišić, Department for Microbiology, Faculty of Veterinary Medicine, University of Belgrade, 11000 Belgrade, Serbia; Department of Functional Food Products Development, Faculty of Biotechnology and Food Science, Wroclaw University of Environmental and Life Sciences, 51-630 Wroclaw, Poland; dusan.misic@upwr.edu.pl

Cedric Muentener, Institute of Veterinary Pharmacology and Toxicology, Vetsuisse-Faculty, University of Zurich, CH-8057 Zurich, Switzerland; cedric.muentener@vetpharm.uzh.ch;

Hanspeter Naegeli, Institute of Veterinary Pharmacology and Toxicology, Vetsuisse-Faculty, University of Zurich, CH-8057 Zurich, Switzerland; hanspeter.naegeli@vetpharm.uzh.ch

Patrizia Nebbia, Pharmacology and Toxicology Department, Faculty of Veterinary Medicine, University of Turin, I-10095 Grugliasco, Turin, Italy; patrizia.nebbia@unito.it

Rosangela Odore, Pharmacology and Toxicology Department, Faculty of Veterinary Medicine, University of Turin, I-10095 Grugliasco, Turin, Italy; rosangela.odore@unito.it

Constança Pomba, CIISA, Centre of Interdisciplinary Research in Animal Health, Faculty of Veterinary Medicine, University of Lisbon, 1300-477 Lisbon, Portugal; cpomba@fmv.ulisboa.pt

Kristi Praakle, Institute of Veterinary Medicine and Animal Sciences, Estonian University of Life Sciences, 51006 Tartu, Estonia; kristi.praakle@emu.ee

Denise Rabold, Institute of Microbiology and Epizootics, Freie Universität Berlin, 14163 Berlin, Germany

Modestas Ružauskas, Microbiology and Virology Institute, Lithuanian University of Health Sciences, LT-44307 Kaunas, Lithuania; modestas.ruzauskas@lsminu.lt

Sara Sacristan, Coordinator Unit of the Spanish National Plan against Antibiotic Resistance, Spanish Agency of Medicines and Medical Devices. 28022 Madrid, Spain; sacristan.sara@gmail.com

Zrinka Štritof, Department of Microbiology and Infectious Diseases with Clinic, Faculty of Veterinary Medicine, University of Zagreb, 10000 Zagreb, Croatia; zstritof@vef.hr

Margarita Terentjeva, Institute of Food and Environmental Hygiene, Faculty of Veterinary Medicine, Latvia University of Life Sciences and Technologies, LV-3004 Jelgava, Latvia; margarita.terentjeva@llu.lv

Marin Torti, Clinic for Internal Diseases, Faculty of Veterinary Medicine, University of Zagreb, 10000 Zagreb, Croatia; titimito@vef.hr

Plamen Trojachanec, Department of Veterinary Surgery, Faculty of Veterinary Medicine, Ss. Cyril and Methodius University, 1000 Skopje, North Macedonia; plament@fvm.ukim.edu.mk

Jaap A. Wagenaar, Department of Infectious Diseases and Immunology, Utrecht University, 3584 CL Utrecht, The Netherlands; j.wagenaar@uu.nl

Irena Zdovc, Institute of Microbiology and Parasitology, Veterinary Faculty, University of Ljubljana, SI-1000 Ljubljana, Slovenia; irena.zdovc@vf.uni-lj.si

* In memoriam

Collaborators Contributions: Resources, N.D.A., A.M.B., D.C., S.C.-M., J.D., M.D., N.F., G.F., L.F., S.V.G., M.G., A.H., S.K., S.S.-L., J.M., D.M., C.M., H.N., P.N., R.O., C.P., K.P., D.R., M.R., S.S., Z.S., M.T., M.T., P.T., J.A.W. and I.Z.; writing—review and editing, N.D.A., A.M.B., D.C., S.C.-M., J.D., M.D., N.F., L.F., S.V.G., M.G., A.H., S.K., S.S.-L., J.M., D.M., C.M., H.N., P.N., R.O., C.P., K.P., D.R., M.R., S.S., Z.S., M.T. (Margarita Terentjeva), M.T. (Marin Torti), P.T., J.A.W. and I.Z.
